# Preconditioning Stimulus Intensity Alters Paired-Pulse TMS Evoked Potentials

**DOI:** 10.3390/brainsci11030326

**Published:** 2021-03-04

**Authors:** Vishal Rawji, Isabella Kaczmarczyk, Lorenzo Rocchi, Po-Yu Fong, John C. Rothwell, Nikhil Sharma

**Affiliations:** 1 Department of Clinical and Movement Neurosciences, Queen Square Institute of Neurology, University College London, London WC1N 3BG, UK; vishal.rawji.11@ucl.ac.uk (V.R.); isabella.kaczmarczyk.17@ucl.ac.uk (I.K.); skgtlro@ucl.ac.uk (L.R.); po-yu.fong.18@ucl.ac.uk (P.-Y.F.); j.rothwell@ucl.ac.uk (J.C.R.); 2 Department of Medical Sciences and Public Health, University of Cagliari, 09124 Cagliari, Italy; 3 Neuroscience Research Center, Chang Gung Memorial Hospital at Linkou, Taoyuan City 333, Taiwan; 4 Division of Movement Disorders, Department of Neurology, Chang Gung Memorial Hospital at Linkou, Taoyuan City 333, Taiwan

**Keywords:** TMS, EEG, TMS-EEG, TEPs, short-interval intracortical inhibition, cortical inhibition, paired-pulse TMS

## Abstract

Motor cortex (M1) paired-pulse TMS (ppTMS) probes excitatory and inhibitory intracortical dynamics by measurement of motor-evoked potentials (MEPs). However, MEPs reflect cortical and spinal excitabilities and therefore cannot isolate cortical function. Concurrent TMS-EEG has the ability to measure cortical function, while limiting peripheral confounds; TMS stimulates M1, whilst EEG acts as the readout: the TMS-evoked potential (TEP). Whilst varying preconditioning stimulus intensity influences intracortical inhibition measured by MEPs, the effects on TEPs is undefined. TMS was delivered to the left M1 using single-pulse and three, ppTMS paradigms, each using a different preconditioning stimulus: 70%, 80% or 90% of resting motor threshold. Corticospinal inhibition was present in all three ppTMS conditions. ppTMS TEP peaks were reduced predominantly under the ppTMS 70 protocol but less so for ppTMS 80 and not at all for ppTMS 90. There was a significant negative correlation between MEPs and N45 TEP peak for ppTMS 70 reaching statistical trends for ppTMS 80 and 90. Whilst ppTMS MEPs show inhibition across a range of preconditioning stimulus intensities, ppTMS TEPs do not. TEPs after M1 ppTMS vary as a function of preconditioning stimulus intensity: smaller preconditioning stimulus intensities result in better discriminability between conditioned and unconditioned TEPs. We recommend that preconditioning stimulus intensity should be minimized when using ppTMS to probe intracortical inhibition.

## 1. Introduction

Transcranial magnetic stimulation (TMS) non-invasively measures motor cortex (M1) function. Paired pulse TMS paradigms (ppTMS) have been used to assess intracortical excitation and inhibition. For example, when a subthreshold conditioning stimulus is delivered before a suprathreshold test stimulus, the motor-evoked potential (MEP) is smaller than from a suprathreshold test stimulus alone. This decrease in MEP amplitude represents corticospinal inhibition and has been termed short-interval intracortical inhibition (SICI) [1,2]. However, as the MEP is a compound signal reflecting cortical, subcortical and spinal excitabilities [3], it is impossible to isolate cortical from non-cortical contributions to the MEP and hence intracortical inhibition. There are several diseases where it would be desirable based on pathophysiological mechanisms to explore the cortical response to a ppTMS without the dependence on the MEP. In this study, we apply a ppTMS paradigm with different conditioning stimuli to healthy volunteers while recording both the MEP and cortical function using EEG. 

Combining TMS and EEG (TMS-EEG) of M1 has the potential to measure cortical function while limiting peripheral confounds. During EEG recording, a TMS stimulus delivered over M1 results in a transient waveform known as the TMS-evoked potential (TEP). The TEP is thought to represent a mixture of several different signals, including, but is not limited to, the physiological response of cortical stimulation, cranial muscle activation artifacts [4] and sensory artifacts from the auditory click of the TMS coil [5]. After removal of muscle and sensory artifacts, a TEP waveform emerges that appears to be largely consistent across different research groups. The TEP after M1 stimulation consists of a number of peaks and troughs that are labelled by their deflection (negative or positive) and the time after TMS stimulus—N15, P30, N45, P55, N100 and P180 [6,7,8,9,10,11,12]. Simultaneous measurement of MEPs has allowed the M1 TEP to be placed in the framework and the existing literature of MEPs and has given insight into the origin of these peaks. Earlier peaks such as the N15 and P30 scale with corticospinal excitability (CSE) [9,13,14], whereas the latter peaks (N45 and P100) are specifically modulated under GABAergic, pharmacologic manipulation [15]. The apparently selective modulation of individual peaks supports that they represent distinct physiological events. Another feature arising from M1 TMS is highly synchronized oscillatory neural activity. In the absence of TMS, EEG and MEG recordings of humans detect this oscillatory activity, which is believed to reflect rhythmic fluctuations in cortical excitability driven by synaptic, intra- and extracellular currents [16]. The functional impact of these fluctuations is most apparent in M1, where stimulation at the peak or trough of beta-frequency oscillatory activity results in larger or smaller values of CSE [17].

Previous studies have combined short-interval ppTMS with EEG [8,13,18,19]. The findings from these studies are inconsistent, potentially owing to differences in methodology such as inter-stimulus interval and stimulation intensities. Most studies [8,13,18] report a reduction in amplitude of P30 (positive deflection occurring 30 ms after the TMS pulse) and P60 (positive deflection occurring 60 ms after the TMS pulse) peaks. Others show either reductions [13] or increases [8,18] of the N45 peak (negative deflection occurring 45 ms after the TMS pulse). On the other hand, Premoli et al. [19] found that this ppTMS paradigm resulted in a marked reduction of N100 and P180 (negative/positive deflections occurring 100/180 ms after the TMS pulse). These changes at N100 and P180 have been attributed to an active, cortical, inhibitory process via GABA_A_ and GABA_B_ receptors. However, the changes in TEP peaks after ppTMS may be the result of a number of different mechanisms unrelated to cortical excitability. As such, we refrain from labelling these as SICI protocols given that the TEP from these protocols has not been clearly defined as arising from the same mechanisms as SICI—instead, we refer to them conservatively as ppTMS protocols. 

A consistent and highly reproducible finding from studying ppTMS and MEPs, is that changing the intensity of the preconditioning stimulus [1,2] alters the balance between excitation and inhibition resulting in a larger or smaller MEPs, respectively. To date, the effect of different preconditioning stimuli on ppTMS has not been studied with TMS-EEG. Here we expand previous work by investigating the effect of different preconditioning stimulus intensities on the TEP waveform evoked by ppTMS. We will place this work in the context of the peripheral (MEP) outcomes that are known to vary with different preconditioning stimulus intensities. We hypothesize that varying the intensity of the preconditioning stimulus to M1 (70–90% resting motor threshold [RMT]) will result in smaller TEP peaks (P30, N45 and P60 peaks) compared to single pulse TMS (spTMS). We also predict that TEP suppression will be greatest when MEP suppression is greatest. Based upon this hypothesis, we specifically focus on EEG electrodes that reflect M1 and will refer to this as a M1 TEP. Given that the MEP and M1 TEP originate from the same stimulus, we hypothesize that TEP peak amplitude will correlate with MEP amplitude. To investigate the effect of ppTMS on oscillatory neural activity we will present the time-frequency characteristics of TEPs during a ppTMS paradigm with different preconditioning stimulus intensities. We expect that the greater synchrony typically seen after a single pulse to be diminished after ppTMS.

## 2. Methods

Preprocessing of the EEG data was performed in MATLAB using the EEGLAB toolbox [20]. Statistical analysis was performed in R (R Core Team, Version 3.5.2, Vienna, Austria).

### 2.1. Participants

15 right-handed subjects (10 female, mean age 24.07, SD 3.79) participated in this experiment. The study was approved by University College London Ethics Committee, in accordance with the Declaration of Helsinki. Inclusion criteria included healthy, consenting adults over the age of 18 of either gender and right-handed. Exclusion criteria included implanted metal objects or devices (cochlear implant or deep brain stimulator) in the brain or skull, taking pro-epileptogenic medication or a history of spinal surgery. No subject had contraindications to TMS, which was assessed by a TMS screening questionnaire. All subjects gave informed consent, provided by a signed information sheet.

### 2.2. Transcranial Magnetic Stimulation and Electromyography Recordings

Throughout the experiment, subjects were seated comfortably in a non-reclining chair, with their forearms supported using a cushion. Electromyographic (EMG) activity was recorded from the right, first dorsal interosseous (FDI) muscle using 19 mm × 38 mm surface electrodes (Ambu WhiteSensor 40713, 2750 Ballerup, Denmark) arranged in a belly tendon montage. The raw signals were amplified, and a bandpass filter was also applied (20 Hz to 2 kHz (Digitimer, Welwyn Garden City, UK)). Signals were digitized at 5 kHz (CED Power 1401; Cambridge Electronic Design, Cambridge, UK) and data were stored on a computer for offline analysis (Signal version 5.10, Cambridge Electronic Design, UK).

Single pulse, monophasic TMS was employed using a Magstim 200^2^ stimulator (The Magstim Co. Ltd., Whitland, Wales) connected via a figure-of-eight coil with an internal wing diameter of 70 mm. A 0.5 cm foam layer was placed underneath the coil to minimize bone conduction of the TMS click and scalp sensation caused by coil vibration. The hotspot was identified as the area on the scalp where the largest and most stable MEPs could be obtained for the right FDI muscle, using a given suprathreshold intensity. The coil was held approximately perpendicular to the presumed left central sulcus and tangentially to the skull, with the coil handle pointing backwards for postero-anterior (PA) stimulation. The RMT was then found with the EEG cap fixed to the head of the subject. This was defined as the lowest TMS stimulus intensity to evoke a response of 50 μV in 5 out of 10 trials in the relaxed FDI using the optimal PA orientation.

Single-pulse TMS (spTMS) was delivered at an intensity required to evoke a motor-evoked potential (MEP) of 1 mV to the left M1 representation of the FDI muscle. This served as a measure of unconditioned CSE. Paired-pulse TMS (ppTMS), was delivered using a preconditioning pulse 2 ms before single-pulse TMS. The intensity of the preconditioning stimulus was changed as a proportion of the RMT (70, 80, 90% of RMT) [21,22]. This resulted in four TMS conditions: spTMS (to measure unconditioned CSE) and three ppTMS paradigms (ppTMS 70, ppTMS 80 and ppTMS 90) to measure corticospinal inhibition. 20 pulses per condition were delivered in a random order during each block, for four blocks. resulting in a total of 320 pulses per session (80 stimuli per condition). Each trial was separated by an inter-trial interval of 3000 ms. The peak-to-peak amplitude of MEPs served as a measure of CSE. Consequently, the decrease in MEP amplitude under ppTMS conditions gives an indication of corticospinal inhibition.

### 2.3. Electroencephalographic Recordings

EEG was simultaneously recorded with TMS using the 64-channel actiChamp System (Brain Products GmbH, Gilching, Germany) in accordance with the 10–20 international EEG electrode array. EEG signals were sampled at 5 kHz and impedances were kept below 5 kΩ. Recordings were referenced to the O_z_ electrode and FP_z_ was made the ground electrode [23]. During online recordings, participants wore earphones covered with headphones, which continuously played white noise. The intensity of this noise was titrated up by the subject to mask the sound of the TMS click and hence reduce any TMS-evoked auditory potentials [24].

TEPs were processed in MATLAB (Version 2017a, MathWorks Inc., Natick, MA, USA) using EEGLAB [20] in conjunction with a series of functions from the TMS-EEG signal analyzer (TESA) toolbox [25]. The TESA toolbox is a specific set of functions designed to analyze TEPs.

Preprocessing was in accordance with the established analysis protocol by Rogasch et al. [25]. Raw EEG signals were epoched (±1.3 s) around the test pulse and demeaned from −1000 ms to −10 ms before the test pulse. Individual trials were visually inspected and rejected if they were particularly noisy, for example, if blinks or eye movements occurred around the TMS pulse or if there were significant drifts in EEG activity preceding the TMS pulse. The TMS artifact was then removed from −5 ms to 15 ms after the time of stimulation, interpolated and then the signal was downsampled to 1000 Hz. A first round of independent component analysis (ICA) using FastICA [26,27] was then performed to remove large, TMS-evoked muscle artifacts (outlined in Rogasch et al. [28]), after which band-pass (1–100 Hz) and band-stop (48–52 Hz) fourth order Butterworth filters were applied. Epoch length was reduced to ±1 s to exclude possible edge artifacts due to filtering. A second round of ICA was then applied to remove residual artifacts based on time, frequency, scalp distribution and amplitude criteria described by Rogasch et al. [25]. Finally, trials were re-referenced to the average reference and binned according to their corresponding trial type for post-processing. All trials, regardless of trial type, were preprocessed together and then separated into their corresponding types (spTMS, ppTMS 70, ppTMS 80 and ppTMS 90). We did this to reduce bias in the preprocessing stage, given the subjective nature of artifact detection, trial omission and independent component selection. On average, 39.3 components were excluded per participant from analysis as they were artifactual in nature (3.8 TMS-evoked muscle, 3.1 eye movement, 25.5 muscle, 6.9 electrode noise). Given that there is the assumption that different trial types can be processed collectively, we also performed our TEP analyses by splitting trials by TMS condition first and preprocessing each independently. 

Local motor cortical excitability was measured by averaging the TEP waveform in a cluster of four electrodes around the site of stimulation (C1, C3, CP1, CP3). This was performed for each condition and the amplitude of characteristic motor TEP peaks (N15, P30, N45, P60, N100 and P180) was calculated [6,7,8,9,10,11,12].

After preprocessing steps, we were left with an average of 73.9 (SD 3.47) trials per condition (spTMS: 74.5, ppTMS 70: 74.6, ppTMS 80: 73.2, ppTMS 90: 73.3).

### 2.4. Data Analysis

#### 2.4.1. MEPs during ppTMS

For each subject, we averaged individual MEPs for each condition, giving an overall indication of CSE for that condition. We performed Wilcoxon signed-rank tests between each ppTMS condition and spTMS MEPs to test for corticospinal inhibition. The increased likelihood of a Type I error occurring as a result of conducting multiple analyses was mitigated with Bonferroni type adjustments.

#### 2.4.2. TEPs during ppTMS

We calculated the average TEP waveform for four electrodes around the site of stimulation (C1, C3, CP1, CP3), for each stimulating condition in each subject. We plotted the mean TEP waveform for all subjects (*n* = 15), for spTMS and ppTMS TMS conditions. Making no assumptions on significance of TEP peaks, we performed a Wilcoxon signed-rank test for timepoints from TMS up to 200 ms after stimulation, whilst correcting for multiple comparisons using the false-discovery rate (FDR) correction method; this resulted in corrections for 200 comparisons.

Given that TEP peaks may represent distinct physiological processes, we extracted the corresponding TEP peak amplitudes from the M1 TEP by finding the peak TEP amplitude in a prespecified window surrounding each time point. The N15 peak was found by finding the negative peak of the TEP between 0 ms and 20 ms after TMS. For P30 this was between 15 ms and 35 ms, N45 between 30 ms and 55 ms, P60 between 50 ms and 70 ms, N100 between 90 ms and 150 ms, and P180 between 150 ms and 250 ms. The polarity of the peak found was in accordance with whether it was a positive or negative defined peak. If no peak was found, then we used the value at that time point (e.g., if no P30 peak was found, the TEP amplitude at 30 ms was used). The absence of peaks occurred in all conditions, albeit in a small number of subjects (spTMS–N15: 2, P30: 5, N45: 5, P60: 1, N100: 0, P180: 1 subjects; ppTMS 70–N15: 1, P30: 1, N45: 3, P60: 1, N100: 1, P180: 0 subjects; ppTMS 80–N15: 1, P30: 2, N45: 3, P60: 2, N100: 1, P180: 0 subjects; ppTMS 90–N15: 2, P30: 3, N45: 4, P60: 1, N100: 0, P180: 0 subjects). We then performed pairwise Wilcoxon signed-rank tests with Bonferroni adjustments between each spTMS TEP peak and each corresponding ppTMS condition TEP peak. Non-parametric tests were used due to the distribution of the data.

#### 2.4.3. The Relationship between the MEP and TEP

In an exploratory analysis, we investigated the relationship between MEPs and TEPs using Spearman’s rank correlation between MEPs and TEP peaks for each ppTMS condition.

#### 2.4.4. Time-Frequency Analysis

TMS-related spectral perturbation (TRSP) and inter-trial coherence (ITC) were computed with EEGLAB for the time-frequency analysis in this study. We analyzed time-frequency characteristics of the same electrodes used in the TEP waveform and peak analyses (C1, C3, CP1, CP3). The time window for TRSP and ITC calculation in each electrode was chosen as 1000 ms before and after the TMS pulse. The baseline period was set from −900 ms to −100 ms. The frequency range for analysis was from 3 Hz to 48 Hz. Sinusoidal wavelet transform was set up as 3 cycles and increased linearly to 24 cycles at 48 Hz. A Hanning-taper window was used. The resolution in frequency was 1 Hz. 200 output time points were generated from −442 ms to 441 ms. The TRSP and ITC were computed based on following equations:

$$TRSP\left(f,t\right)=\frac{1}{n}\overset{n}{\underset{k=1}{\sum }}|F_{k}\left(f,t\right){|}^{2}$$

$$ITC\left(f,t\right)=\frac{1}{n}\overset{n}{\underset{k=1}{\sum }}\frac{F_{k}\left(f,t\right)}{|F_{k}\left(f,t\right)|}$$

In the equations, the trial k at frequency f and time t could have the spectral estimate $F_{k}\left(f,t\right)$ when total n trials [20,29].

Paired t test by Monte Carlo permutation test with 1000 random partitions was applied to compare TRSP and ITC between ppTMS and spTMS conditions [30]. Cluster correction was used for the multiple comparisons and the threshold for statistical significance was set to 0.05. 

### 2.5. Data Availability

Data and code will be deposited in an appropriate repository once accepted for publication.

## 3. Results

### 3.1. Physiological Measurements

Mean RMT and 1 mV intensity was measured at 53.9% (SD 8.2) and 64.4% (SD 9.0) of the maximum stimulator output, respectively. 

### 3.2. The Effect of ppTMS on MEPs

**Figure 1:**
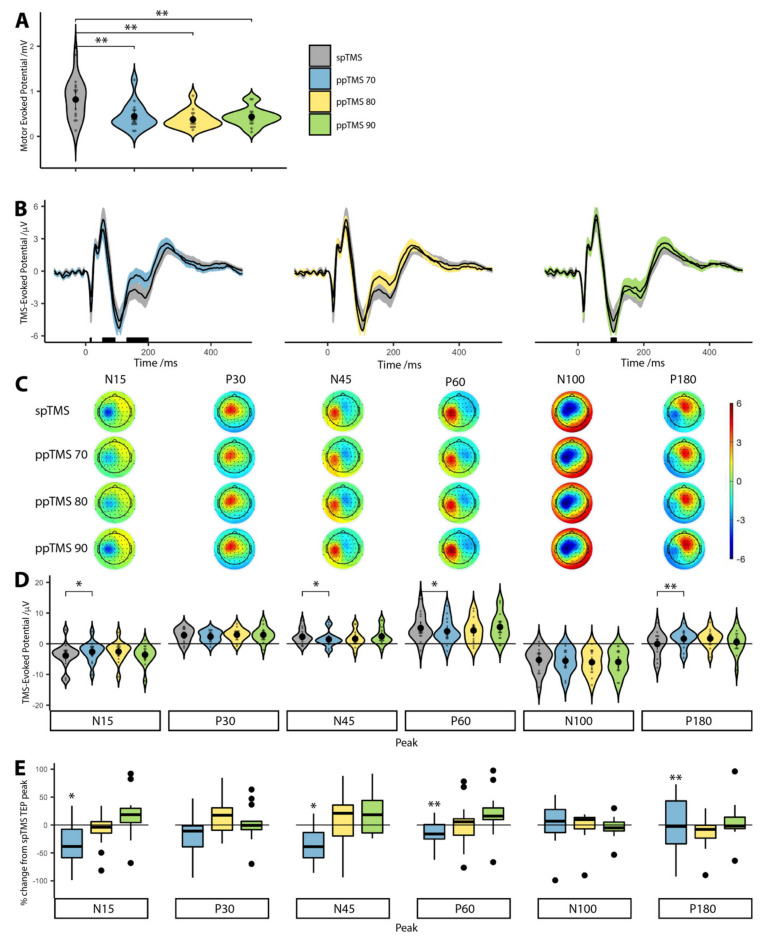
Corticospinal and cortical inhibition measured during ppTMS protocols. (**A**): Corticospinal excitability measured by MEP amplitudes after spTMS and each of the ppTMS conditions. (**B**): Average TEP waveform from four electrodes around the site of stimulation (C1, C3, CP1 and CP3). Shaded areas around the TEP waveform represent standard error of the mean. Black bars under each plot represent statistically significant (p < 0.05) time points, calculated from Wilcoxon signed-rank tests, after FDR correction. (**C**): Scalp plots showing the TEP distribution across our six timepoints of interest, for each condition. (**D**): TEP peaks at six time-points (15 ms, 30 ms, 45 ms, 60 ms, 100 ms and 180 ms) are extracted for each subject and plotted for each stimulation condition. (**E**): Percentage change of individual TEP peaks during ppTMS conditions with respect to spTMS TEPs. Due to differences in polarity between conditions, values were squared first and then represented as a fraction of the spTMS TEP peak. Values below the dashed line depict suppression; those above depict facilitation. Asterisks represent statistically significant differences (* = *p* < 0.05, ** = *p* < 0.01).

Bonferroni corrected Wilcoxon signed-rank tests showed that median CSE was significantly lower than spTMS (0.82 mV) for ppTMS 70 (0.37 mV, *p* = 0.068), ppTMS 80 (0.32 mV, *p* = 0.008) and ppTMS 90 (0.43 mV, *p* = 0.052). These results confirmed that CSE was suppressed during each ppTMS protocol (Figure 1A).

### 3.3. The Effect of ppTMS on M1 TEPs

FDR corrected Wilcoxon signed-rank tests showed that only the ppTMS 70 TEP waveform differed significantly from the spTMS TEP waveform (Figure 1B) at approximately 15 ms, 60 ms and 180 ms. Similarly, we observed topography-wide differences in cortical activity after ppTMS (Figure 1C). We also note that this effect is still present when TEPs are analyzed by splitting trials by condition prior to pre-processing (Supplementary Figure S1). 

Median ppTMS 70 TEP test ranks were statistically different than the median spTMS TEP ranks for the N15 (Z = −2.04, *p* = 0.041), N45 (Z = −2.36, *p* = 0.018), P60 (Z = −2.64, *p* = 0.008) and P180 (Z = −2.93, *p* = 0.003) peaks. The P30 (Z = −0.47, *p* = 0.639) and N100 (Z = −0.64, *p* = 0.524) peak ranks did not differ statistically from spTMS TEP peaks.

For ppTMS 80 there was a trend towards a difference for the N15 (Z = −1.79, *p* = 0.073), N100 (Z = −1.67, *p* = 0.095) and P180 (Z = −1.92, *p* = 0.055) peaks. There were no statistically significant rank differences for the remaining peaks (P30: Z = −0.25, *p* = 0.804; N45: Z = −1.55, *p* = 0.121; P60: Z = −1.14, *p* = 0.252). 

For ppTMS 90, there were no statistically significant median rank differences (N15: Z = −0.08, *p* = 0.934; P30: Z = −0.25, *p* = 0.639; N45: Z = −0.14, *p* = 0.890; P60: Z = −0.14, *p* = 0.890; N100: Z = −1.32, *p* = 0.188 and P180: Z = −0.64, *p* = 0.524). As we included the amplitude even when a peak was not present, we re-analyzed the data including only peaks; the results did not significantly alter.

We normalized ppTMS TEP peak amplitudes to spTMS conditions, to compare percentage changes of individual peak amplitudes at different ppTMS conditions (Figure 1E), which showed similar results to analysis of the non-normalized peak data (Figure 1D). 

We conclude that despite MEP suppression for all three ppTMS conditions, the TEP did not follow the same pattern. TEP peaks were reduced predominantly under the ppTMS 70 protocol but less so for ppTMS 80 and ppTMS 90.

### 3.4. Relationship between MEPs and TEPs

**Figure 2:**
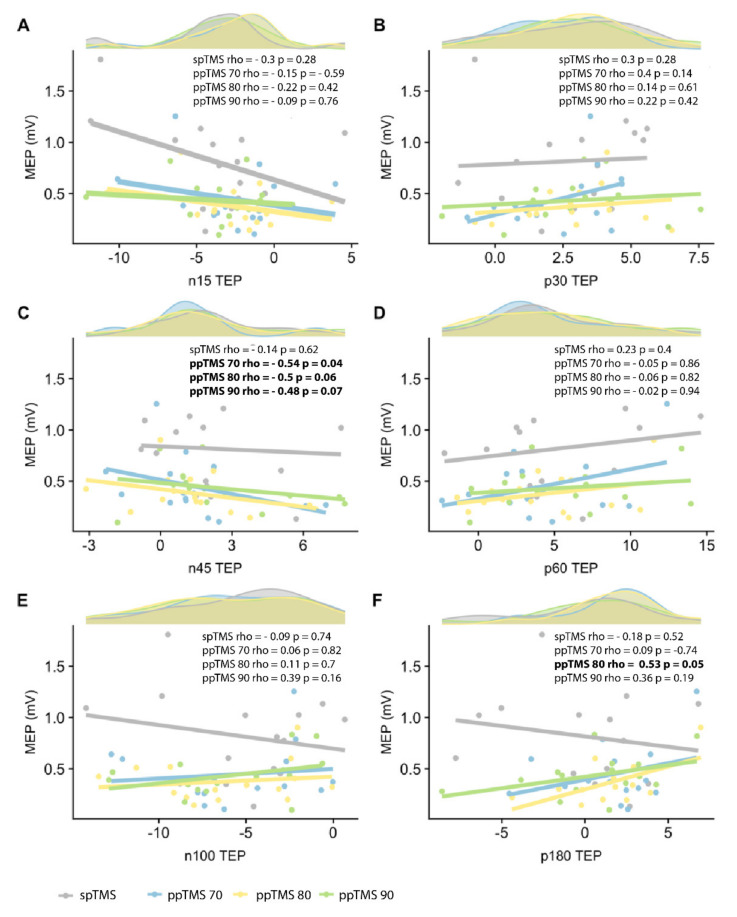
Spearman rank correlations between MEP and TEP peaks for single pulse, ppTMS 70, 80, 90. Each plot (**A**–**F**) shows Spearman rank correlations between the MEP amplitude and TEP peak amplitude (N15, P30, N45, P60, N100 and P180), for spTMS and each ppTMS condition. Above each plot is the distribution of each peak per condition. Time-frequency characteristics of ppTMS TEPs. Bold text in the figure represents interactions reaching statistical significance at a level of 0.05.

To explore the relationship between MEPs and TEPs, we performed Spearman’s rank correlation coefficients between MEPs and TEP peaks for each ppTMS condition (Figure 2). There was a significant negative correlation between MEPs and N45 TEP peak for ppTMS 70 (rho = −0.54, *p* = 0.04). The same trend was maintained across ppTMS 80 and 90 (ppTMS 80: rho = −0.5, *p* = 0.06; ppTMS 90: rho = −0.48, *p* = 0.07). There was a positive correlation between MEPs and P180 for ppTMS 80 (rho = 0.53, *p* = 0.05). 

**Figure 3:**
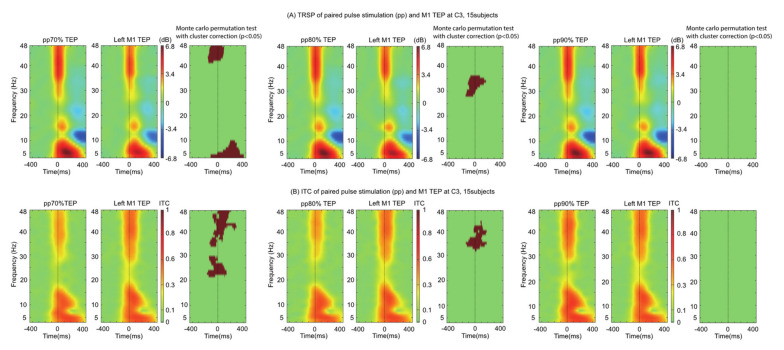
TEP-related spectral perturbation (TRSP) and intertrial coherence (ITC) of spTMS and ppTMS 70, 80, 90 conditions for the C3 electrode. Top row (**A**) shows the 5-48 Hz TRSP with warmer colors depicting greater power and cooler colors depicting lower power. Bottom row (**B**) shows ITC, where values vary from 0 to 1 (1 represents perfect coherence between trials, whereas 0 represented no coherence between trials). Monte Carlo permutation tests with cluster correction are performed and shown adjacent to TRSP/ITC plots. Threshold for statistical significance is set to 0.05. Each ppTMS condition is paired with the spTMS (Left M1 TEP) condition to facilitate visualization.

We explored the time-frequency characteristics of ppTMS TEPs by comparing them to spTMS TEPs. The TRSP was found to be smaller for both ppTMS 70 (5–10 Hz and 40–48 Hz) and ppTMS 80 (28–36 Hz) conditions, but not ppTMS 90. The ppTMS ITC showed a similar pattern: ITC differences were more widespread for ppTMS 70 (20–48 Hz) than for ppTMS 80 (31–42 Hz) and ppTMS 90. In all, the results mirrored those found with ppTMS TEP waveforms and peaks: there was a dependence of time-frequency measures on preconditioning stimulus intensity (Figure 3).

## 4. Discussion

We show that the M1 TEP after ppTMS is smaller in amplitude than when the M1 TEP is unconditioned [8,13,18,19], reaching significance for ppTMS 70 condition only. We expand the previous literature by showing that the change in M1 TEP due to ppTMS is dependent on the intensity of the preconditioning stimulus: the smaller the preconditioning stimulus intensity, the greater the discriminability between conditioned and unconditioned TEPs. 

During ppTMS 70, there is significant inhibition of the N15, N45, P60 and P180 TEP peaks. Our findings are supported by the previous literature, particularly modulation of the N45 [8,13,18], which was significantly correlated with the degree of corticospinal inhibition. The earliest observable peak in our study, the N15, has been suggested to reflect activation of the posterior parietal cortex, following M1 TMS [8,31]. N45 peak modulation scales with CSE suggesting it could be a reflection of M1 excitability. In fact, dipole modelling suggests that the origin of the N45 peak is likely to be M1 [11]. The P60 peak, which was also modulated, is thought to reflect general cortical excitability [9,13,14]. In all, a decrease in cortical excitability seems to mediate the decrement in TEP amplitude following ppTMS. 

Whilst M1 TEPs were significantly smaller during the ppTMS 70 protocol, this was not the case for the other stimulation protocols (ppTMS 80 and ppTMS 90), contrasting with the simultaneously measured MEPs. This is affirmed by the time-frequency analysis that demonstrates lower synchronization with smaller preconditioning stimuli during the ppTMS paradigm (Figure 3). There are a number of possible explanations for this.

First, the approach to analysis and underlying assumptions of MEPs and TEPs are very different. MEP analysis requires few steps, with peak-to-peak amplitude measurements of an amplified signal using surface EMG electrodes. Conversely, TEP analysis has several steps such as filtering, noise reduction and independent component analysis. While we followed the steps for a previously established TEP analysis pipeline [25,28], each step has a number of assumptions that manipulates the data. While the underlying process may be the same, the difference in the approaches may alter the output and mask the effects of cortical inhibition from TEP analysis. 

Oscillatory activity induced by TMS is largely consistent across different stimulating conditions (Figure 3) and induces widespread activity spanning many different frequency bands, an observation which has previously been characterized [32]. Whilst motor cortical activity is dominated by activity in the beta frequency, it is interesting to note that the changes relative to ppTMS occupy the gamma, delta and theta frequencies. This discrepancy may reflect the recruitment of inhibitory interneurons during intracortical inhibition. Alternatively, it may be the case that the preconditioning stimulus oscillatory activity ‘interferes’ with the test pulse evoked activity resulting in a smaller TEP. It is conceivable, therefore, that the evoked oscillatory activity of two different pulses would result in more ‘interference’ whereas two similar pulses would result in less ‘interference’. In other words, when the test and conditioning pulses are markedly different (i.e., 70% RMT and 120% RMT) the resulting TEP is smaller—which is supported by our data. Whilst the difference between 70% and 80% RMT represents a small absolute difference in stimulator output, cortical reactivity is non-linear. Consequently, a small absolute change in conditioning stimulus intensity may result in a large cortical response, which is closer in resemblance to that produced by the test stimulus. What about the juxtaposed position? We explored this in an additional experiment when the stimuli are matched (70% RMT × 70% RMT ppTMS) and found that there was no difference in the TEPs (*n* = 8) providing some support for this view.

Unlike EEG, surface EMG data represents a highly filtered signal. The EEG electrode configuration uses 64 electrodes to record cortical activity; TEPs represent a composite signal of cortico-cortical activity whereas MEPs measure corticospinal activity for a particular muscle [32]. TMS recruits a heterogenous mixture of neural elements, including excitatory and inhibitory interneurons, and even interneurons not relevant to the FDI [33]; EEG does not differentiate between these different sources, whereas the FDI MEP represents a somatotopically sensitive output. In other words, the FDI MEP represents a highly filtered signal. In fact, the MEP is a smoothed average of descending cortical output onto spinal motor neurons [3]. 

The M1 TEP, on the other hand, is an unfiltered signal (is less topographically selective for the FDI) composed of activity relevant to the FDI cortical motor neurons and other neurons that are not our primary focus. Consequently, increasing preconditioning stimulus intensity increasingly results in activation of neurons not relevant to the FDI axis and this extra neural noise (i.e., not FDI relevant) contributes to the TEP but not the MEP. Hence, whilst the FDI MEP may be inhibited, the TEP does not discriminate FDI related cortical activity from non-FDI related cortical activity. With increasing preconditioning stimulus intensities, the amount of this extra neural noise increases and the discrimination between spTMS and ppTMS TEPs, decreases. This argument is strengthened when taking into consideration that increasing TMS intensity increases recruitment of excitatory interneurons related to facilitatory intracortical processes [34]. In the study by Peurala and colleagues, there was a shift from intracortical inhibition to intracortical facilitation as TMS preconditioning stimulus intensity increases, presumably due to contamination from the recruitment of excitatory interneurons. We propose that this is why ppTMS 70 TEPs are statistically different to spTMS TEPs, whereas ppTMS 80 and 90 are not. Indeed, TEP peaks were more strongly correlated with MEP size for ppTMS conditions with smaller preconditioning stimuli, supporting the hypothesis that increasing the preconditioning stimulus results in additional noise in the EEG signal that is not reflected in the MEP. 

A popular method for inferring the physiological relevance of M1 TEPs has been to investigate how these TEPs and their features covary with MEPs, primarily due to the wealth of prior MEP research. A key finding from the present study is that noise can be introduced to the TEP signal with increasing stimulus intensities, which makes inferences based on MEPs more difficult. Going forward, we recommend that the intensity of preconditioning stimuli should be minimized when attempting to measure ppTMS TEPs using TMS-EEG for two primary reasons: (1) increasing stimulus intensity activates neurons not relevant to the FDI axis, and (2) increasing stimulus intensity activates neurons not relevant to intracortical inhibition. 

ppTMS has the ability to probe excitatory and inhibitory intracortical circuits. In doing so, ppTMS has found clinical potential in neurological disorders affecting cortical regions [35,36]. For example, MEPs measured using ppTMS have been proposed as an objective marker of cortical function in amyotrophic lateral sclerosis (ALS) [37]. However, this approach suffers from conceptual flaws preventing its clinical translation. As MEPs are a composite measure of upper and lower motor neurons, their amplitude may be confounded by the lower motor neuron degeneration found in ALS [38]. To this end, a TMS-EEG approach would circumnavigate the lower motor neuron contribution and hence provide a cortical measure of M1 function [39].

## 5. Limitations

TEPs have been considered to include sensory feedback from muscle activation, which may contaminate TEP measurements [5]. Whilst this is a caveat to the interpretation of TEPs, the latency of sensory feedback (auditory) affects later portions of the TEP beyond 100 ms [40,41] and are largely confined to the auditory cortex. In fact, selective modulation of early TEP components and corticospinal excitability has been shown with transcranial direct current stimulation, showing that early TEP components are more likely to be reflective of M1 TMS and less likely to be influenced by sensory contamination [41]. In addition the event-related desynchronisation due to afferent proprioceptive information, has been observed after 300 ms post-stimulation [32]. Consequently, the findings of peaks preceding 100 ms (N15, P30, N45 and P60 peaks) remain unaffected by the confound of sensory feedback. We identified 70% RMT as the optimal preconditioning stimulus intensity to measure ppTMS-TEPs. However, this suggestion is constrained by the range of preconditioning stimuli used in the present study, which were chosen based on optimal preconditioning stimulus intensities for SICI measured using MEPs. Indeed, it may be the case that a preconditioning stimulus with lower intensity may be better at differentiating spTMS and ppTMS TEPs. Future work should aim to explore the full range of preconditioning stimulus intensities during ppTMS. 

## 6. Conclusions

As previously reported, we show that the TEP generated after ppTMS is smaller than that generated by spTMS. However, this finding depends on the intensity of the preconditioning stimulus, such that using smaller preconditioning stimuli in ppTMS protocols results in greater discriminability of the TEP decrement. The dissociation between peripheral (MEP) and cortical (TEP) measures of ppTMS suggest that ppTMS TEPs may not reflect the same cortical, inhibitory process underlying the decrement in ppTMS MEPs.

## Data Availability

The data and code used to generate the results in this paper are available upon reasonable request to the corresponding author, N.S.

## Supplementary Materials

The following are available online at https://www.mdpi.com/2076-3425/11/3/326/s1, Figure S1: TEP waveforms analyzed after splitting by condition prior to pre-processing.
