# Supplementary material for: Preconditioning Stimulus Intensity Alters Paired-Pulse TMS Evoked Potentials
Source: Brain Sci. 2021 Mar 4;11(3):326. doi: 10.3390/brainsci11030326 (PMC7998341; doi:10.3390/brainsci11030326)
Supplement: Supplementary file 1 [file brainsci-11-00326-s001.pdf]

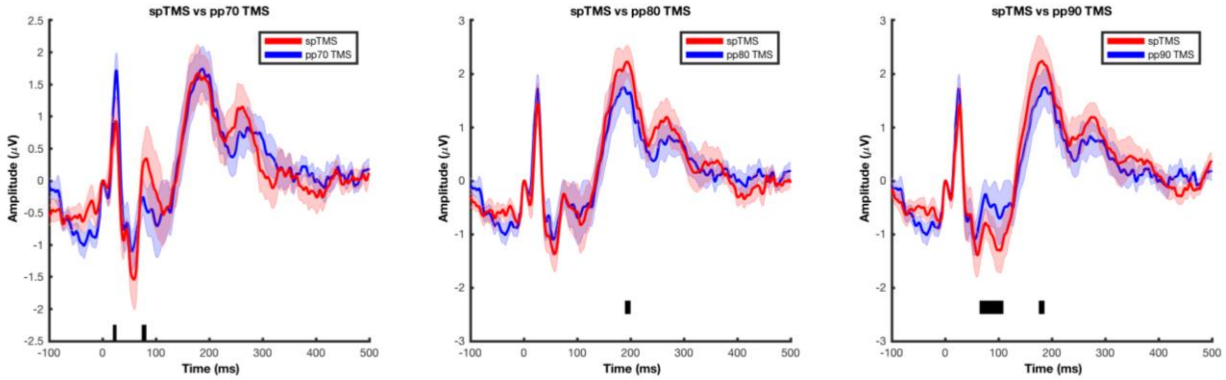


**Supplementary figure 1: TEP waveforms analyzed after splitting by condition prior to pre-processing.**

Figures show TEP waveforms, which use a different pre-processing pipeline to that described in the main manuscript. Here, trials are split by condition first, given the hypothetical differences in TMS-induced artifacts from paired-pulse stimuli vs single-pulse stimuli. Pre-processing, including two rounds of ICA, are performed on each of the split datasets, rather than performing ICA on the whole dataset and splitting by trials after. Plots show spTMS, and pp70, pp80 and pp90TMS TEPs. Black bars show areas of FDR-corrected statistical significance at an alpha of 0.05.
